# HIV Denial in the COVID Era

**DOI:** 10.1007/s10461-024-04528-3

**Published:** 2024-10-12

**Authors:** Tara C. Smith

**Affiliations:** College of Public Health, Kent State University, Kent, USA Ohio

**Keywords:** Misinformation, Denial, Disinformation, Social media, Science communication

## Abstract

Though scientific consensus regarding HIV causation of AIDS was reached decades ago, denial of this conclusion remains. The popularity of such denial has waxed and waned over the years, ebbing as evidence supporting HIV causation mounted, building again as the internet facilitated connection between denial groups and the general public, and waning following media attention to the death of a prominent denier and her child and data showing the cost of human life in South Africa. Decades removed from these phenomena, HIV denial is experiencing another resurgence, coupled to mounting distrust of public health, pharmaceutical companies, and mainstream medicine. This paper examines the history and current state of HIV denial in the context of the COVID pandemic and its consequences. An understanding of the effect of this phenomenon and evidence-based ways to counter it are lacking. Community-based interventions and motivational interviewing may serve to contain such misinformation in high-risk communities.

## Introduction

In 2022, AIDS (Acquired Immune Deficiency Syndrome) was responsible for approximately 630,000 deaths globally [1]. Since its identification in 1981, it has been estimated that more than 81 million people have been infected with the Human Immunodeficiency Virus (HIV), the virus that causes AIDS [2]. In the 40 years since the discovery of the virus, we have made progress in understanding its molecular biology and pathogenesis and have developed a number of successful pharmaceutical interventions to prevent new infections or treat those already infected. HIV infection has moved from an almost certain death sentence in the 1980s to a manageable chronic condition today.

Despite overwhelming evidence collected confirming HIV as the causative agent of AIDS since the recognition of the virus in 1983, there is still denial in some conspiracist circles regarding AIDS causation (reviewed in [3]). Though HIV denial, broadly defined as the belief that HIV does not cause AIDS, appears to be a relatively fringe conspiracy theory in 2024, it has experienced a minor revival in recent years. HIV denial has merged with vaccine and germ theory denial in the wake of the pandemic, most notably connected by 2024 presidential candidate Robert F. Kennedy Jr. in his 2021 book, “*The Real Anthony Fauci*” [4]. Between publication of the book and various media interviews and social media posts, Kennedy has repeated conspiracy theories about vaccines, HIV, and bioweapons development in multiple interviews and panels.

As such, the current state of HIV denial persists within a field of broad-scale denial of many aspects of infectious disease epidemiology and biology more generally. Here, I examine the history and present situation of HIV denial, with a focus on Kennedy’s claims and how they have encouraged others to revisit old conspiracy theories, and discuss what may be used to counter this misinformation.

## The Early History of HIV Denial, 1980s–1990s

The early years of the HIV pandemic were understandably confusing, where innuendo and rumor filled the voids created by scientists’ lack of knowledge regarding viral epidemiology and pathogenesis. Our understanding of HIV and of AIDS as a disease were in its infancy; the general public was fearful of contact with individuals who were HIV positive as clear answers regarding transmission were still being developed. Stigma was rife and treatments were not yet available. As such, it was the perfect environment for rumors and incomplete knowledge to morph into active doubt and misinformation. Though he was not the first to cast doubt regarding HIV causation of AIDS, the beginning of a proper HIV denial movement is often traced to the 1987 publication of an article by molecular biologist Peter Duesberg [5] in which he wrote, “It is concluded that AIDS virus is not sufficient to cause AIDS and that there is no evidence, besides its presence in latent form, that it is necessary for AIDS.”

The 1987 paper later became the first chapter in a collection Duesberg published as a 1995 book, “*Infectious AIDS: Have We Been Misled?*” [6]. Collectively, these papers still form the basis of many HIV denial arguments, which were further developed in the 1996 publication “*Inventing the AIDS Virus*,” [7], a more layperson-friendly book.

Duesberg’s reputation as a member of the National Academy of Sciences and professor at the University of California, Berkeley provided him with a megaphone for his claims. More importantly, his background and position gave such claims immediate scientific credibility, even when they contrasted with ongoing work by colleagues actively studying the epidemiology or molecular biology of HIV/AIDS. Most in the scientific field merely ignored Duesberg’s publications [8] as he moved farther from the scientific mainstream.

By 1991, Duesberg was part of a group formally requesting a re-evaluation of the HIV/AIDS hypothesis, known as The Group for the Scientific Reappraisal of the HIV/AIDS Hypothesis (currently “Rethinking AIDS”). In addition to Duesberg, this group was comprised of several lawyers including Phillip E. Johnson, also a prominent denier of evolutionary theory; journalists Celia Farber and Tom Bethell (another evolution denier); and molecular biologists Harvey Bialy and Charles A. Thomas Jr. in addition to others. A second group of deniers based in Australia, the Perth Group, also denied HIV causation of AIDS but went a step farther by claiming that HIV does not exist at all. Their website states “Due to irreconcilable scientific and ethical differences we disassociate ourselves from the Rethinking AIDS Group.” As such, even deniers of HIV causation cannot agree on the basic tenets of HIV biology.

A 1994 Science article discussed what was dubbed the “Duesberg Phenomenon” [8], with author Jon Cohen spending 3 months interviewing Duesberg’s supporters and detractors as well as examining the AIDS literature of the time. Cohen noted, “Most AIDS researchers thought Duesberg was exploiting uncertainties about the precise mechanism of disease causation to discount a mountain of compelling epidemiologic, laboratory, and animal data supporting the conclusion that HIV causes AIDS. But the press was less skeptical,” [8] which allowed HIV denial to grow, amplified by sympathetic journalists such as Bethell and Farber.

## HIV Denial in the Early 2000s

By the late 1990s and early part of the 21st century, combination therapies which included newly-discovered protease inhibitors led to more effective treatment for and prevention of HIV infection [9]. The films *Philadelphia* and *And the Band Played On*, both released in 1993, put HIV/AIDS in the public spotlight. Animal models of infection and the accidental infection of laboratory workers and dental patients appeared to fulfill Koch’s postulates, a key Duesberg criticism [10]. Researchers assumed HIV denial would “fade away” [11]. Instead, the early 2000s saw a minor resurgence in HIV denial in US popular culture, with reverberations around the globe. Celia Farber re-emerged with an influential 15-page article in Harper’s Magazine, “Out of Control: AIDS and the Corruption of Medical Science” [12]. Though scientists pointed out the article’s “glaring errors” [13, 14], HIV researcher Seth Kalichman noted it “represented a breakthrough of HIV/AIDS denialism into mainstream media” [15].

Perhaps the most influential new addition to the AIDS denial movement was Christine Maggiore, the author of “*What if Everything You Thought You Knew About AIDS was Wrong?*” [16] and the founder of “Alive and Well AIDS Alternatives” where Peter Duesberg served as an advisory board member. Maggiore tested HIV-positive in 1992 [17]. Initially, she volunteered to speak as an HIV educator with Project AIDS Los Angeles until meeting with Duesberg in 1994, eventually deciding that her HIV test was a false positive. She did not take any drugs for herself even when pregnant and breastfeeding her two children, who were also unvaccinated [17], and focused on convincing other HIV-positive women to avoid testing and HIV drugs during and after pregnancy. Her husband, Robin Scovill, shared her views, writing, directing, and producing a 2004 film starring Duesberg and Maggiore, “*The Other Side of AIDS*.” Given her wealthy Los Angeles background, Maggiore also recruited Foo Fighters bassist Nate Mendel to her cause, speaking before a 2000 concert that benefited Alive and Well [18]. The Foo Fighters also provided songs for the soundtrack for Scovill’s film.

Globally, Duesberg and Maggiore were both influences on South African president Thabo Mbeki. In May 2000, Mbeki invited Duesberg and other denialists to “debate” the virus along with HIV scientists and clinicians [19]; Duesberg was one scientist chosen to represent the views of HIV denialists. Mbeki spoke with Maggiore at the 13th International AIDS Conference in Durban, South Africa in July, 2000 [20] during a time when Mbeki and his health minister were debating use of AZT (azidothymidine) [21], the first medication approved to prevent or treat HIV infection and to reduce the likelihood of transmission from an infected pregnant person to the fetus during pregnancy. Scientists found Mbeki’s doubtful stance so alarming that they released the “Durban declaration” [22], a declaration signed by over 5,000 scientists and physicians affirming that HIV is the cause of AIDS. The eventual cost of Mbeki’s denial was profound: estimates suggest that because interventions that existed at the time were not employed, 330,000 lives were lost, and 35,000 babies were born with HIV between 2000 and 2005 in South Africa [23].

Maggiore’s daughter, Eliza Jane, died of AIDS-related pneumonia at age 3 in 2005 [17]. Negative publicity from Eliza Jane’s death ensued, including an appearance by Maggiore on ABC’s Primetime Live in 2005 and a “ripped from the headlines” episode of “*Law & Order: Special Victims Unit*” reflecting the case. Maggiore herself died in 2008, reportedly from AIDS-associated complications [24]. With the death of Maggiore and her daughter and the public backlash to HIV denial, the movement lost momentum.

## Modern HIV Denial: Merger into all-purpose Science Denial

Since the start of the COVID pandemic, trust in public health has declined [25] while vaccine hesitancy rose [26]. In both cases, the changes were driven by individuals who identify as Republican [27], though trust in science declined in those on the left as well. Such distrust was promoted by individuals such as Robert F. Kennedy Jr. Kennedy was once a respected environmental lawyer and activist, but for decades has made broad claims that undermine confidence in vaccines and in response for all infectious diseases more broadly. In 2005, he debuted his anti-vaccine views in the essay “*Deadly Immunity*” [28], published jointly in Rolling Stone magazine and online at Salon. Within days of publication, multiple errors were pointed out and corrected by Salon [29], undermining the story’s message and Kennedy’s credibility. Salon retracted the article in 2011 [29].

In 2007, Kennedy founded the non-profit World Mercury Project to fight against environmental aspects Kennedy believed were detrimental to health, including mercury, pesticides, and fluoride. Over time, the organization’s targets included vaccines, changing the name to Children’s Health Defense (CHD) in 2018. In 2021, the Center for Countering Digital Hate included Kennedy and CHD in their list of the “disinformation dozen”—the 12 influencers responsible for two-thirds of anti-vaccine misinformation on social media [30]. Since then, he has not only continued to spread anti-vaccine misinformation, but has been described as promoting “an alternative worldview firmly anchored in conspirituality” [31]. This branched out to other infectious disease conspiracy theories, including the idea that the origins of HIV and the 1918 influenza pandemic came from “vaccine research” [32, 33]. Kennedy stated, “The medical research on these diseases and vaccine research has actually created some of the worst plagues in our history. Anybody who reads ‘*The River*’ will come away pretty much convinced that HIV also came from a vaccine program, there’s plenty of evidence on that as well.” “*The River*” was a 1999 book by Edward Hooper [34] which suggested that HIV originated from contaminated monkey cells used for polio vaccine research and testing [35–37]. Epidemiological and molecular data debunked that allegation [38, 39], but the rise of charges of COVID origins as a “lab leak” [40] has brought such ideas back into view.

The merger of vaccine and pandemic denial was bolstered with the 2021 publication of Kennedy’s “*The Real Anthony Fauci*” [4], which is rife with tactics and tropes common in science denial [3, 41–43] (see Table 1). Kennedy begins Chap. 5, “*The HIV Heresies*,” saying he hesitated to include the chapter “because any questioning of the orthodoxy that HIV is the sole cause of AIDS remains an unforgivable—even dangerous—heresy among our reigning medical cartel and its media allies.” Though he claims to “take no position on the relationship between HIV and AIDS,” the book quotes HIV deniers approvingly over hundreds of pages, including Duesberg and Christine Maggiore, whom he never notes died of AIDS along with her daughter. He later states in a parenthetical, “(For the record, I believe that HIV is a cause of AIDS, but Dr. Fauci’s acknowledgment of non-HIV AIDS shows that causation is more complex than the official theology).”

**Table 1:** Common science denial tropes and their use in “The Real Anthony Fauci”

Trope [3, 41–43]	Example quotations from “The Real Anthony Fauci” [4]
Portraying science and faith and consensus as dogma	“It is a hazard to both democracy and public health when a kind of religious faith in authoritative pronouncements supplants disciplined observation, rigorous proofs, and reproducible results as the source of ‘truth’ within the medical field.” (pg. 178) “Instead of responding to critics by answering common-sense inquiries, Dr. Fauci has cultivated a theology that denounces questioning his orthodoxy as irresponsible, uninformed, and dangerous heresy.” (p. 179) “Suspending traditional skepticism toward government pronouncements, the press ordained Gallo’s theory as indisputable doctrine and beatified Gallo as a saint.” (pg. 184) “Dr. Fauci summoned the entire upper clergy of his HIV orthodoxy—and all of its lower acolytes and altar boys—to unleash a storm of fierce retribution on the Berkeley virologist and his followers.” (p. 214)
Supporters of scientific consensus are merely defending Big Pharma/ “pharma shill” accusation	“Duesberg and others charge that by stifling debate and dissent, Dr. Fauci milled public fear into multi-billion-dollar profits for his Pharma partners while expanding his own powers and authoritarian control.” (p. 180) “PIs [Principal investigators] are pharmaceutical industry surrogates who play key roles promoting the pharmaceutical paradigm and functioning as high priests of all its orthodoxies, which they proselytize with missionary zeal.” (p. 136) “Dr. [Paul] Offit serves on the board of various pharma front groups and astroturf organizations and commands a vast network of bloggers and trolls, each of them directly or indirectly paid by the pharmaceutical companies to stifle debate, propagate lies, bully and intimidate the mothers of intellectually disabled children, silence scientific and medical dissent, and root out heresy.” (p. 138–139)
**Claim of increasing support**	“I have learned that today, a disturbing number of virologists quietly doubt the theory that HIV is the sole cause of AIDS.” (p. 181)
Cherry-picking evidence	“During the thirty-six years since Dr. Fauci and his colleague, Dr. Robert Gallo, first claimed that HIV is the sole cause of AIDS, no one has been able to point to a study that demonstrates their hypothesis using accepted scientific proofs.” (pg 178–179)
Deathbed confession	“On his deathbed, the victorious Pasteur is said to have recanted, ‘Béchamp was right,’ declaring, ‘the microbe is nothing. The terrain is everything.’” (p. 286)
Science is settled by debate	“The fact that Dr Fauci has obstinately refused to describe a convincing scientific basis for his proposition, or to debate the topic with any qualified critics, including the many Nobel laueates who have expressed skepticism, makes it even more important to give air and daylight to dissenting voices.” (p. 179) “Dr. Fauci’s control of his PI army gives him the ability to shut down all debate. When National Public Radio attempted to stage a conversation between Duesberg and a supporter of the HIV hypothesis, it could find no one willing to confront him.” (p. 218) “When [Harvey] Bialy challenged Dr. John Moore of Cornell University to a debate on AIDS, Moore wrote in reply: “Participating in any public forum with the likes of Bialy would give him a credibility that he does not merit.” (pg. 218)

Like the HIV denial groups mentioned above, Kennedy can’t make up his mind about the existence of HIV itself, criticizing HIV scientist Robert Gallo’s delayed release of a blood test Kennedy claims resulted in “thousands of hospital patients and hemophiliacs received tainted blood from blood banks and became infected with HIV,” yet states just a few pages later that “…it is unclear whether Gallo or any other researcher was ever able to isolate HIV”. The same arguments are used today regarding SARS-CoV-2, with deniers suggesting alternatively that (1) it does not exist and tests are merely picking up cellular debris; (2) it does exist and was made as a bioweapon in a Chinese laboratory; but (3) that despite its development as a “bioweapon,” it is a harmless, “common cold” virus.

Kennedy even flirts with outright germ theory denial, discussing “terrain theory,” the idea that if one’s body is health enough, it cannot be damaged by microbes (referring to it as “miasma theory”). He explains:

Germ theory aficionados, in contrast, blame disease on microscopic pathogens. Their approach to health is to identify the culpable germ and tailor a poison to kill it. Miasmists complain that those patented poisons may themselves further weaken the immune system, or simply open damaged terrain to a competitive germ or cause chronic disease…When a starving African child succumbs to measles, the miasmist attributes the death to malnutrition; germ theory proponents (a.k.a. virologists) blame the virus.

**Fig. 1:**
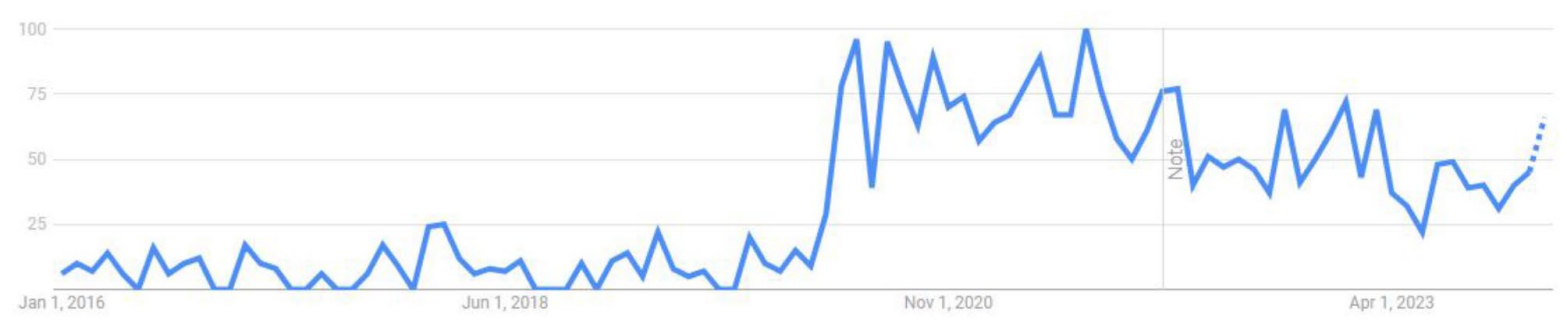
Searches for “Terrain theory” from 2016 to 2024, via Google Trends. A marked increase is seen at the start of the COVID pandemic, which remains above pre-pandemic levels through early 2024

Such germ theory denial has also become more public during the pandemic [44]. Google searches for “terrain theory” increased in March 2020 and have remained relatively high since, compared to the 4 years prior to the pandemic (Fig. 1).

The pandemic also brought new relevance and interest to extant groups that had seen their message diminished in recent years, including Rethinking AIDS, who wrote of COVID [45]:

Fast forward nearly forty years to Covid-19, and what does one find? The same scare tactics, the same contradictions, the same wrong predictions, the same bogus tests, the same deadly treatment approach, all this but more global, more coordinated, and even more socially destructive. And the General leading the charge? Anthony Fauci.

A central function of the new Rethinking AIDS/Unmasking Covid is to expose for all to see, this profound relationship between Covid-19 and HIV/AIDS. As the original AIDS dissidents, we are uniquely equipped for the task.

They refer to COVID as “the new AIDS”, suggesting that coronavirus does not cause COVID, and that COVID was a plot “as its goal the manufacture and sale of vaccines” and “an attempt to impose a whole new socio-economic and socio-political structure onto the rest of the world” [46]. Kennedy even brought back journalist Celia Farber as a guest on CHD TV’s “*The People’s Testament*” online videocast, discussing parallels between what she describes as “medical murder pertaining to the AIDS epidemic” and the COVID response [47].

The publishing landscape during the pandemic also reflects a possible resurgence in HIV denial. In addition to Kennedy’s publication, Simon & Schuster distributed a 2023 book by HIV denialist Rebecca Culshaw, “*The Real AIDS Epidemic: How the Tragic HIV Mistake Threatens Us All*” [48], a barely-updated version of her 2007 book “*Science Sold Out: Does HIV Really Cause AIDS?*” [49]. Following pushback from scientists, medical professionals, and activists regarding the release of her book [50], Culshaw penned a follow-up ebook on Amazon, “*Almost Cancelled Part One: How AIDS activists almost stopped the publication of The Real AIDS Epidemic*” [51], noted to be “part one of a series of forthcoming books on the subject of censorship in AIDS”.

Previous research has shown that vaccine denial can easily lead to belief in other conspiracy theories, and vice versa [52]. Beyond Kennedy, vaccine denier Steve Kirsch has railed against fluoride [53] while football player Aaron Rodgers, once rumored to be a potential Kennedy running mate who spread misinformation about vaccines and COVID [54], reportedly shared Sandy Hook conspiracy theories [55]. Influencers who were instrumental in spreading COVID misinformation, such as podcaster Joe Rogan [56] and biologist Bret Weinstein [57] have also promoted HIV denial [58, 59]. Tropes used by science deniers of any type follow a very similar pattern which emphasize distrust of the government and scientific authority; portrayal of science as faith and consensus as dogma; naturalistic fallacies suggesting anything “natural” is good but “manmade” is harmful; and “skewing the science” by cherry-picking low-quality evidence in support of their position while rejecting higher-quality studies that show vaccine safety and efficacy [3, 42, 43, 60], see Table 1.

With COVID, we also saw the rise of “medical freedom” groups [61]. These groups had previously existed to oppose vaccine mandates for school entry, growing through the 2010s as states such as California, Maine, and New York reduced non-medical exemptions to vaccination. Many “medical freedom” advocates quickly pivoted from anti-vaccine activism to include opposition to the mask mandates and other restrictions that were instituted during the early years of the pandemic. Researcher Peter Hotez noted that “antivaccine and anti-COVID-19 prevention blended with political extremism on the far right, and even with QAnon and other conspiracy-laden movements” [61]. Politicization of vaccination hesitancy and resistance has long been a concern [42], and this has materialized during the pandemic [62]. It is unclear where HIV denial currently sits along political boundaries.

## What does Cause AIDS, if not HIV?

Incredibly, the answer to this remains unchanged from the early 1980s, and once again most responses trace back to Duesberg. Kennedy describes the ideas of Duesberg and Mullis regarding AIDS causation as belief in:

a multiplicity of environmental exposures that became ubiquitous in the 1980s. The HIV virus, this group insists, was a kind of free rider that was also associated with overlapping lifestyle exposures. Duesberg and many who have followed him offered evidence that heavy recreational drug use in gay men and drug addicts was the real cause of immune deficiency among the first generation of AIDS sufferers. They argued that the initial signs of AIDS, Kaposi’s sarcoma and *Pneumocystis carinii* pneumonia (PCP), were both strongly linked to amyl nitrate—‘poppers’—a popular drug among promiscuous gays. [4].

After 1987, Duesberg argued that in addition to illicit drugs, many AIDS deaths were actually caused by AZT, and that “HIV does not cause AIDS but is simply a ‘free rider’ common to high-risk populations who suffer immune suppression due to environmental exposures”. AIDS deaths in developing countries are said to be merely diseases such as tuberculosis and malaria, re-labeled.

## Discussion

Improvements have been made in HIV treatment over the last three decades, moving from the initial nucleoside reverse transcriptase inhibitors such as AZT to modern-day combination therapies, which are highly effective and reduce the risk of viral drug resistance [63]. Such anti-HIV therapies have converted HIV infection into a manageable chronic condition, where those infected can expect to have a greatly improved quality of duration of life compared to those living with HIV in the 1980s and 1990s. For those with HIV who have access to HAART, life expectancy is now almost the same as the general population [64], and treatment can be received in as little as an injection of two medicines once every two months [65].There certainly have been disappointments as well, including the failure to develop an effective vaccine, equity of access to HIV testing and treatment, and lingering stigma.

While climate change denial is primarily a right-wing phenomenon [66] and anti-vaccination sentiment has moved rightward since the pandemic [62], HIV denial has historically spanned the political spectrum. In 1994, Science journalist Jon Cohen noted Duesberg supporters within the gay community in the San Francisco Castro district, as well as conservatives who have “little sympathy for the gay movement” and individuals who “believe the establishment is always wrong” [8]. Conservative and support of HIV denial included the “anti-state, anti-war, pro-market” Lew Rockwell blog, which is host to hundreds of science denial posts including “Fallacies in Modern Medicine: HIV/AIDS” [67], “Why I Quit HIV” [68] by Rebecca Culshaw, and “My HIV Investigation, and parallels to the COVID Hoax” [69]. Conservative publishing house Regnery has published books by Duesberg and Bethell [7, 70]. But the left side of the political spectrum has also promoted HIV denial. Left-leaning Mothering magazine, “the magazine of natural family living,” published a cover article denying HIV in 2001 and encouraging HIV-positive mothers to birth “outside the system” in order to avoid interventions that lessen the risk of HIV transmission to the infant [71]. Kennedy’s Presidential run initially began as a Democrat, but moved to an Independent ticket late in 2023. He suspended his campaign to endorse Republican Donald Trump in August 2024, claiming he will be “deeply involved in helping to choose the people who can run F.D.A. and N.I.H. and C.D.C.” in the event of a Trump victory [72].

As there are still many gaps in our knowledge of the effects of HIV misinformation, there are no validated best practices to specifically counter HIV/AIDS denial. Debunking false claims via fact-checking or rebuttals is common (e.g [73])., but not necessarily effective [74]. It should be noted that denial does not always fade as overwhelming evidence against it mounts. Sociologist Keith Kahn-Harris notes, “Central to denialism is an argument that ‘the truth’ has been suppressed by its enemies” [75]; as such, factual information, no matter how voluminous, can be discarded as false out of hand if it comes from untrusted sources. Unlike attitudes regarding climate change and vaccination, there are few representative surveys of the general public asking about beliefs regarding HIV/AIDS, so it is difficult to ascertain how far this misinformation permeates into the general population and who is most susceptible. It is also unclear how much influence such ideas have on receipt of HIV treatment or prevention. A 2004 study found that 41% of self-reported HIV-positive men who have sex with men (MSM) agreed, at least somewhat, that HIV does not cause AIDS [76]. A 2005 study examining conspiracy beliefs and condom use in African Americans found that in men, higher level of belief in HIV/AIDS conspiracy theories was related to lower consistent use of condoms [77]. As these studies primarily focus on African-Americans, general medical mistrust may act in concert with specific HIV rumors. Such misinformation can also percolate from individuals such as Duesberg and others and spread into the mainstream via celebrities or other influencers, including Will Smith and Dave Chappelle [78].

Extrapolation could potentially be made from other types of denial, especially vaccine hesitancy which shares many features. To encourage vaccination among the vaccine hesitant, many clinics already work with local leaders who are trusted within communities to help provide accurate information and work with healthcare providers to assure testing, treatment, and prevention, and to address any rumors or misinformation that may be reducing one’s intent to receive testing or other services. Care must be taken not to minimize legitimate concerns, and to respond to queries with empathy [78]. Use of motivational interviewing, while labor-intensive, may be an important strategy to address concerns about HIV/AIDS rumors in infected or at-risk populations [79, 80]. Finally, lessons can be drawn from studies examining corrective responses to conspiracy theories more broadly [81], including different types of “pre-bunking,” where individuals are taught misinformation techniques in order to inoculate them against such messaging in the future [82, 83].

## References

[1] El-Sadr WM, Cohen MS. Global fight against HIV is at risk. Science. 2023;382:621. 37917748 10.1126/science.adm6975

[2] UNAIDS. Fact sheet - Latest global and regional statistics on the status of the AIDS epidemic, 2023.

[3] Smith TC, Novella SP. HIV denial in the Internet era. PLoS Med. 2007;4:e256. 17713982 10.1371/journal.pmed.0040256 PMC1949841

[4] Kennedy Jr. RF. The real Anthony Fauci: Bill Gates, Big Pharma, and the Global War on Democracy and Public Health. New York: Skyhorse; 2021.

[5] Duesberg PH. Retroviruses as carcinogens and pathogens: expectations and reality. Cancer Res. 1987;47:1199–220. 3028606

[6] Duesberg PH, Infectious AIDS. Have we been misled? Berkeley, CA: North Atlantic Books; 1995.

[7] Duesberg PH. Inventing the AIDS Virus. Gateway Books; 1996.

[8] Cohen J. The Duesberg phenomenon. Science. 1994;266:1642–4. 7992043 10.1126/science.7992043

[9] Volberding PA. An aggressive approach to HIV antiretroviral therapy. Hosp Pract (1995). 1998;33:81–47. 9464233 10.1080/21548331.1998.11443622

[10] O’Brien SJ, Goedert JJ. HIV causes AIDS: Koch’s postulates fulfilled. Curr Opin Immunol. 1996;8:613–8. 8902385 10.1016/s0952-7915(96)80075-6

[11] Moore J, Nattrass N. Deadly Quackery. The New York Times. New York, New York, 2006.

[12] Farber C. Out of control: AIDS and the corruption of Medical Science. Harper’s Magazine; 2006.

[13] Miller L. An Article in Harper’s Ignites a Controversy Over H.I.V. The New York Times. New York, New York, 2006:5.

[14] Gallo R, Geffen N, Gonsalves G et al. Accessed September 5. Errors in Celia Farber’s March 2006 article in Harper’s Magazine. https://www.natap.org/2006/HIV/ErrorsInFarberArticle.pdf. 2024.

[15] Kalichman SC, Denying AIDS. Conspiracy theories, pseudoscience, and human tragedy. New York: Copernicus Books; 2009.

[16] Maggiore C. What if Everything You Thought you knew about AIDS was wrong? American Foundation For Aids Alternatives; 2000.

[17] Ornstein C, Costello D. A mother’s denial, a daughter’s death. California: Los Angeles Times. Los Angeles; 2005.

[18] Talvi S. Foo fighters, HIV deniers. Mother Jones; 2000.

[19] Sidley P. Mbeki appoints team to look at cause of AIDS. BMJ. 2000;320:1291. 10807605 PMC1127296

[20] Yamey G. HIV disinformation. West J Med. 2000;173:300–1. 11069856 10.1136/ewjm.173.5.300 PMC1071141

[21] Cherry M. AZT critics ‘swayed South African president’. Nature. 1999;402:225. 10580484 10.1038/46140

[22] The Durban Declaration. Nature. 2000;406:15–6. 10894520 10.1038/35017662

[23] Chigwedere P, Seage GR 3rd, Gruskin S, Lee TH, Essex M. Estimating the lost benefits of antiretroviral drug use in South Africa. J Acquir Immune Defic Syndr. 2008;49:410–5. 19186354 10.1097/qai.0b013e31818a6cd5

[24] Gorman A, Zavis A. Christine Maggiore, vocal skeptic of AIDS research, dies at 52. Los Angeles, California: Los Angeles Times; 2008.

[25] Pollard MS, Davis LM. Decline in Trust in the Centers for Disease Control and Prevention during the COVID-19 pandemic. Rand Health Q. 2022;9:23.35837520

[26] Kuzelewska E, Tomaszuk M. Rise of conspiracy theories in the pandemic Times. Int J Semiot Law. 2022;35:2373–89. 35910405 10.1007/s11196-022-09910-9 PMC9325658

[27] Kennedy B, Tyson A. Americans’ trust in scientists, positive views of Science continue. to Decline: Pew Research Center; 2023.

[28] Kennedy Jr. RF. Deadly immunity. Rolling Stone; 2005.

[29] Lauerman K. Correcting our record. Salon, 2011.

[30] CDDH. The Disinformation Dozen: Why Platforms Must Act on Twelve Leading Online Anti-Vaxxers, 2021.

[31] Jarry J. I watched a Week’s Worth of RFK Jr.’s fear-inducing TV Channel. Canada: McGill Office for Science and Society. Montreal; 2023.

[32] Merlan A, Robert F. Kennedy Jr. Gives the Game Away. Vice, 2023.

[33] Ramirez NM, Klee M, RFK Jr. Claims ‘Vaccine Research’ likely responsible for HIV and the spanish flu. Rolling Stone; 2023.

[34] Hooper E. The river: a journey to the source of HIV and AIDS. Boston, MA: Little Brown & Co; 1999.

[35] Elswood BF, Stricker RB. Polio vaccines and the origin of AIDS. Med Hypotheses. 1994;42:347–54. 7935079 10.1016/0306-9877(94)90151-1

[36] Elswood BF, Stricker RB. Polio vaccines and the origin of AIDS. Res Virol. 1993;144:175–7. 8511401 10.1016/s0923-2516(06)80027-0

[37] Cohen J. Debate on AIDS origin: Rolling Stone weighs in. Science. 1992;255:1505. 1549779 10.1126/science.1549779

[38] Worobey M, Santiago ML, Keele BF, et al. Origin of AIDS: contaminated polio vaccine theory refuted. Nature. 2004;428:820. 15103367 10.1038/428820a

[39] Koprowski H. My response to polio vaccines and the origin of AIDS. Res Virol. 1995;146:233–4. 7481096 10.1016/0923-2516(96)80584-x

[40] Lenharo M, Wolf L. US COVID-origins hearing renews debate over lab-leak hypothesis. Nature. 2023;615:380–1. 36890328 10.1038/d41586-023-00701-1

[41] Kata A. Anti-vaccine activists, web 2.0, and the postmodern paradigm–an overview of tactics and tropes used online by the anti-vaccination movement. Vaccine. 2012;30:3778–89. 22172504 10.1016/j.vaccine.2011.11.112

[42] Smith TC. Vaccine rejection and hesitancy: a review and call to action. Open Forum Infect Dis. 2017;4:ofx146. 28948177 10.1093/ofid/ofx146 PMC5597904

[43] Smith TC, Reiss DR. Digging the rabbit hole, COVID-19 edition: anti-vaccine themes and the discourse around COVID-19. Microbes Infect. 2020;22:608–10. 33171267 10.1016/j.micinf.2020.11.001 PMC7648494

[44] Gorski D. Germ theory denial in the age of the COVID-19 pandemic, 2021.

[45] DiFerdinando T, Rethinking AIDS. Accessed February 6. https://rethinkingaids.com/index.php/introduction. 2024.

[46] DiFerdinando T. Accessed February 6. Introductory remarks from the new President of Rethinking AIDS, Tom DiFerdinando, at the Covid-19: the New AIDS conference. https://rethinkingaids.com/index.php/introduction/president-s-opening-remarks-to-covid-the-new-aids-conference. 2024.

[47] Defense CsH. Murder by Medicine. The People’s Testament. Children’s Health Defense; 2023.

[48] Culshaw RV. The Real AIDS Epidemic: How the Tragic HIV Mistake Threatens Us All. Skyhorse, 2023.

[49] Culshaw RV. Science sold out: does HIV really cause AIDS? North Atlantic Books; 2007.

[50] Saha J. Public health advocates demand Simon & Schuster stop distribution of AIDS denialism book. Salon, 2023.

[51] Culshaw RV. Almost Cancelled Part One: How AIDS activists almost stopped the publication of The Real AIDS Epidemic, 2023:89.

[52] Bensley DA, Lilienfeld SO, Rowan KA, Masciocchi CM, Grain F. The generality of belief in unsubstantiated claims. Appl Cogn Psychol. 2020;34:16–28.

[53] Kirsch S. The Case Against Fluoride. Steve Kirsch’s newsletter. Vol. 2024. Substack.com, 2022.

[54] Belson K, Anthes E. Scientists Fight a New Source of Vaccine Misinformation: Aaron Rodgers. The New York Times. New York, New York, 2021:7.

[55] Brown P, Tapper J. RFK Jr.’s VP prospect Aaron Rodgers has shared false Sandy Hook conspiracy theories in private conversations. CNN, 2024.

[56] Qui L. Fact-checking Joe Rogan’s interview with Robert Malone that caused an uproar. New York, NY: The New York Times; 2022.

[57] Effinger A. A Progressive Biologist from Portland is one of the Nation’s leading advocates for Ivermectin. Willamette Week; 2021.

[58] Rogan J. The Joe Rogan Experience. Bryan Callen: #282–Peter Duesberg; 2012.

[59] Rogan J. The Joe Rogan Experience. #2101 Bret Weinstein; 2024.

[60] Sadaf A, Richards JL, Glanz J, Salmon DA, Omer SB. A systematic review of interventions for reducing parental vaccine refusal and vaccine hesitancy. Vaccine. 2013;31:4293–304. 23859839 10.1016/j.vaccine.2013.07.013

[61] Hotez PJ. America’s deadly flirtation with antiscience and the medical freedom movement. J Clin Invest 2021; 131:e149072. 10.1172/JCI149072 PMC8011881 33630759

[62] Bolsen T, Palm R, Politicization. COVID-19 vaccine resistance in the U.S. Prog Mol Biol Transl Sci. 2022;188:81–100. 35168748 10.1016/bs.pmbts.2021.10.002 PMC8577882

[63] Gibas KM, Kelly SG, Arribas JR, et al. Two-drug regimens for HIV treatment. Lancet HIV. 2022;9:e868–83. 36309038 10.1016/S2352-3018(22)00249-1 PMC10015554

[64] Trickey A, Sabin CA, Burkholder G, et al. Life expectancy after 2015 of adults with HIV on long-term antiretroviral therapy in Europe and North America: a collaborative analysis of cohort studies. Lancet HIV. 2023;10:e295–307. 36958365 10.1016/S2352-3018(23)00028-0 PMC10288029

[65] Pebody R, Hayes R. Accessed September 4. What do we know about injectable HIV medication? https://www.aidsmap.com/about-hiv/what-do-we-know-about-injectable-hiv-medication. 2024.

[66] Hamilton LC, Hartter J, Lemcke-Stampone M, Moore DW, Safford TG. Tracking Public Beliefs about Anthropogenic Climate Change. PLoS ONE. 2015;10:e0138208. 26422694 10.1371/journal.pone.0138208 PMC4589389

[67] Miller Jr. DW. Fallacies in Modern Medicine. HIV/AIDS; 2014.

[68] Culshaw R, Why I, Quit HIV. Lew Rockwell Blog, 2006.

[69] Rappoport J, My HIV. Investigation, and parallels to the COVID hoax. L:ew Rockwell Blog; 2021.

[70] Bethell T. The Politically Incorrect Guide to Science. Regnery, 2005.

[71] Gerhard S. Safe and Sound Underground: HIV-Positive women birthing outside the system. Mothering Magazine; 2010.

[72] Bedard R. What to Expect if R.F.K. Jr. Is Promoted to a Position of Power. The New York Times. New York, New York, 2024.

[73] Campaign TA, Debunking AIDS, Denialism. Accessed September 5 https://www.tac.org.za/news/debunking-aids-denialism/. 2024.

[74] Chan MS, Jones CR, Hall Jamieson K, Albarracin D. Debunking: a Meta-analysis of the psychological efficacy of messages countering misinformation. Psychol Sci. 2017;28:1531–46. 28895452 10.1177/0956797617714579 PMC5673564

[75] Kahn-Harris K, Denialism. What Drives People to Reject the Truth. The Guardian 2018.

[76] Hutchinson AB, Begley EB, Sullivan P, Clark HA, Boyett BC, Kellerman SE. Conspiracy beliefs and trust in information about HIV/AIDS among minority men who have sex with men. J Acquir Immune Defic Syndr. 2007;45:603–5. 17704688 10.1097/QAI.0b013e3181151262

[77] Bogart LM, Thorburn S. Are HIV/AIDS conspiracy beliefs a barrier to HIV prevention among African americans? J Acquir Immune Defic Syndr. 2005;38:213–8. 15671808 10.1097/00126334-200502010-00014

[78] Heller J. Rumors and realities: making sense of HIV/AIDS conspiracy narratives and contemporary legends. Am J Public Health. 2015;105:e43–50. 25393166 10.2105/AJPH.2014.302284 PMC4265931

[79] Corwin MA. Motivational interviewing and HIV: a guide for navigators. In: NMAC, ed.

[80] Gagneur A. Motivational interviewing: a powerful tool to address vaccine hesitancy. Can Commun Dis Rep. 2020;46:93–7. 32281992 10.14745/ccdr.v46i04a06 PMC7145430

[81] Prike T, Ecker UKH. Effective correction of misinformation. Curr Opin Psychol. 2023;54:101712. 37944323 10.1016/j.copsyc.2023.101712

[82] van der Linden S, Roozenbeek J. Inoculation Resist Misinformation JAMA. 2024;331:1961–2. 38753337 10.1001/jama.2024.5026

[83] Cook J, Lewandowsky S, Ecker UKH. Neutralizing misinformation through inoculation: exposing misleading argumentation techniques reduces their influence. PLoS ONE. 2017;12:e0175799. 28475576 10.1371/journal.pone.0175799 PMC5419564

